# Networking to implement diagnostic capacity and (re-) evaluate the public health importance of leptospirosis in the Institut Pasteur International Network

**Published:** 2011-01-10

**Authors:** P Boisier, F Berger, P Bourhy, B Guillard, S Guyomard-Rabenirina, K Kouadio, M Picardeau, C Goarant

**Affiliations:** 1 Centre Pasteur du Cameroun, Yaounde, Cameroon; 2 Institut Pasteur de Guyane, 97306 Cayenne Cedex, French Guiana; 3 Institut Pasteur, Paris, France; 4 Centre National de Référence, Institut Pasteur, Paris, France; 5 Institut Pasteur du Cambodge, Phnom Penh, Cambodia; 6 Institut Pasteur de Guadeloupe, Abymes, Guadeloupe; 7 Institut Pasteur de Cote d’Ivoire, Abidjan, Ivory Coast; 8 Institut de Nouvelle Calédonie, Nouméa, New Caledonia

## 

Leptospirosis is a tropical neglected disease that is highly under-diagnosed and under-reported, mostly because of a lack of diagnosis capacity. The Institut Pasteur International Network includes researchers and physicians that have a recognized expertise in the field of leptospirosis epidemiology and diagnosis. Taking advantage of this international and widespread network of expertise, we currently aim at implementing the diagnosis of leptospirosis in institutes, countries or regions where it was not available. With the financial support of the Institut Pasteur International Network, biologists and researchers from French Guyana, Cameroon, Guadeloupe (West Indies), Cote d’Ivoire, Cambodia, New Caledonia and France currently work at implementing and inter-calibrating leptospirosis diagnosis in their respective institutes.

Because of very slow and delicate culture of *Leptospira* spp., the early diagnosis of leptospirosis increasingly relies on PCR. Using its own molecular platform and expertise, each institute implements the PCR or qPCR method of its choice for the detection of pathogenic *Leptospira* spp. Common *Leptospira* suspensions are shared to commonly evaluate the sensitivity of the PCR assays.

The serological reference method uses the Micro Agglutination Test (MAT), a method that requires continuous culture of live leptospires and experienced staff for interpretation. Therefore, commercially available serological tests (Elisa for the detection of anti-Leptospira IgM) are used as a first diagnosis approach. The French National Reference Center then validates the results and identifies the infecting serogroup using a reference MAT panel.

These diagnostic tools are then offered to patients hospitalized with predefined leptospirosis-like symptoms, as a way to (re-) evaluate the contribution of leptospirosis to non-malarial febrile illnesses in these countries and regions.

The global architecture and goals of our Lepto-network will be presented, together with epidemiological research perspectives using the tools and expertise developed through this network.

